# Membrane property and biofunction of phospholiposome incorporated with anomeric galactolipids

**DOI:** 10.1186/s40064-016-2236-z

**Published:** 2016-05-17

**Authors:** Danyang Liu, Junqi Zhang, Shouhong Xu, Honglai Liu

**Affiliations:** Key Laboratory for Advanced Materials and Institute of Fine Chemicals, School of Chemistry and Molecular Engineering, East China University of Science and Technology, 130 Meilong Rd, Shanghai, 200237 People’s Republic of China; Key Laboratory of Medical Molecular Virology (Ministry of Health and Ministry of Education), School of Basic Medical Sciences, Fudan University, 138 Yixueyuan Rd, Shanghai, 200032 People’s Republic of China

**Keywords:** Glycolipid, Hybrid liposome, Interfacial behaviour, DNA condensation

## Abstract

There has been increasing interest in the construction of liposomes containing a targeting reagent for target-specific drug delivery. Glycoconjugates that can be recognized by transmembrane glycoprotein receptors have been extensively used to form glyco-liposomal drug carriers. However, the impact of anomerism, which is a common identity of natural glycoconjugates, on the glyco-liposomal properties has been hardly probed in previous studies. Here we investigate the liposomal properties of phospholipid incorporated with a pair of anomeric galactolipids. The anomeric galacto-liposomes are characterized and their membrane fluidity, thermo-stability, DNA condensation efficiency and fluorescence leakage are comparatively tested. The in vitro cellular internalization effect of the galacto-liposomes is also demonstrated. This study suggests that anomerism might give distinct impact on the membrane properties and even biofunctions of glyco-liposomes.

## Background

Liposome has the similar structure to biomembrane, such as cell membrane, and is always used as substitute in research of biomembrane. It is easy to believe that the properties of membranes should give large influence on their biofunctions. For example, lower thermal stability might increase membrane permeability and release drug quickly (Yang et al. 2014). Positive charged liposomal surface might promote interaction with cells (Rädler et al. 1997). And even, the membrane fusion should be determined partly by properties of bilayer membranes.

In the drug or DNA delivery system, liposomes can effectively protect cargoes from being biodegraded in vivo before reaching a target tissue. However, the drug delivering efficiency of commercial liposomes is usually compromised due to the lack of a ‘warhead’ that effectively directs them to the target. In recent years, construction of glycolipid (a saccharide covalently linked to a lipid)-incorporated liposomes (Jayaraman et al. 2013; Ueno et al. 2007; Róg et al. 2007; Ramezani et al. 2009; Stimac et al. 2012; Yin et al. 2013) has been of increasing interest since the glycosyl group of which can be specifically recognized by a transmembrane glycoprotein receptor of a target tissue (Weis et al. 1998; Marth and Grewal 2008). The recognition may then facilitate the endocytosis and/or fusion of the liposomes by the host cells, releasing an encapsulated therapeutic agent. Among them, galactoliposomes that can be recognized by the asialoglycoprotein receptors (ASGPR) expressed by the hepatocyte (Rigopoulou et al. 2012) are the most extensively developed, and are considered to be promising non-viral vectors for target-specific treatment of hepatic diseases (Sliedregt et al. 1999; Zhao et al. 2011; Symens et al. 2012; Hu et al. 2013; Jain et al. 2012). Monosaccharides are linked to each other or with an aglycon in either the α- or β-configuration (at the anomeric carbon) to form cell-surface ‘antennas’, such as glycoproteins or glycolipids, that transmit signals between cells (Varki 1993; Bertozzi and Kiessling 2001; Seeberger 2005; Hart and Copeland 2010). These anomeric counterparts may, however, possess different physiochemical attributes, thereby leading to a diversification of biological function. In addition, some synthetic anomeric glycolipids have shown distinct pharmacological properties (He et al. 2011). Despite the fruitful results obtained in the investigation of glycolipid-based liposomal transfection (Ueno et al. 2007), to our knowledge, investigations on the properties of anomeric galacto-liposomes have been elusive.

**Fig. 1:**
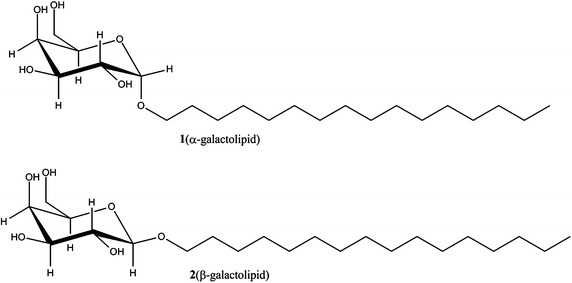
Structure of anomeric galactolipids

**Fig. 2:**
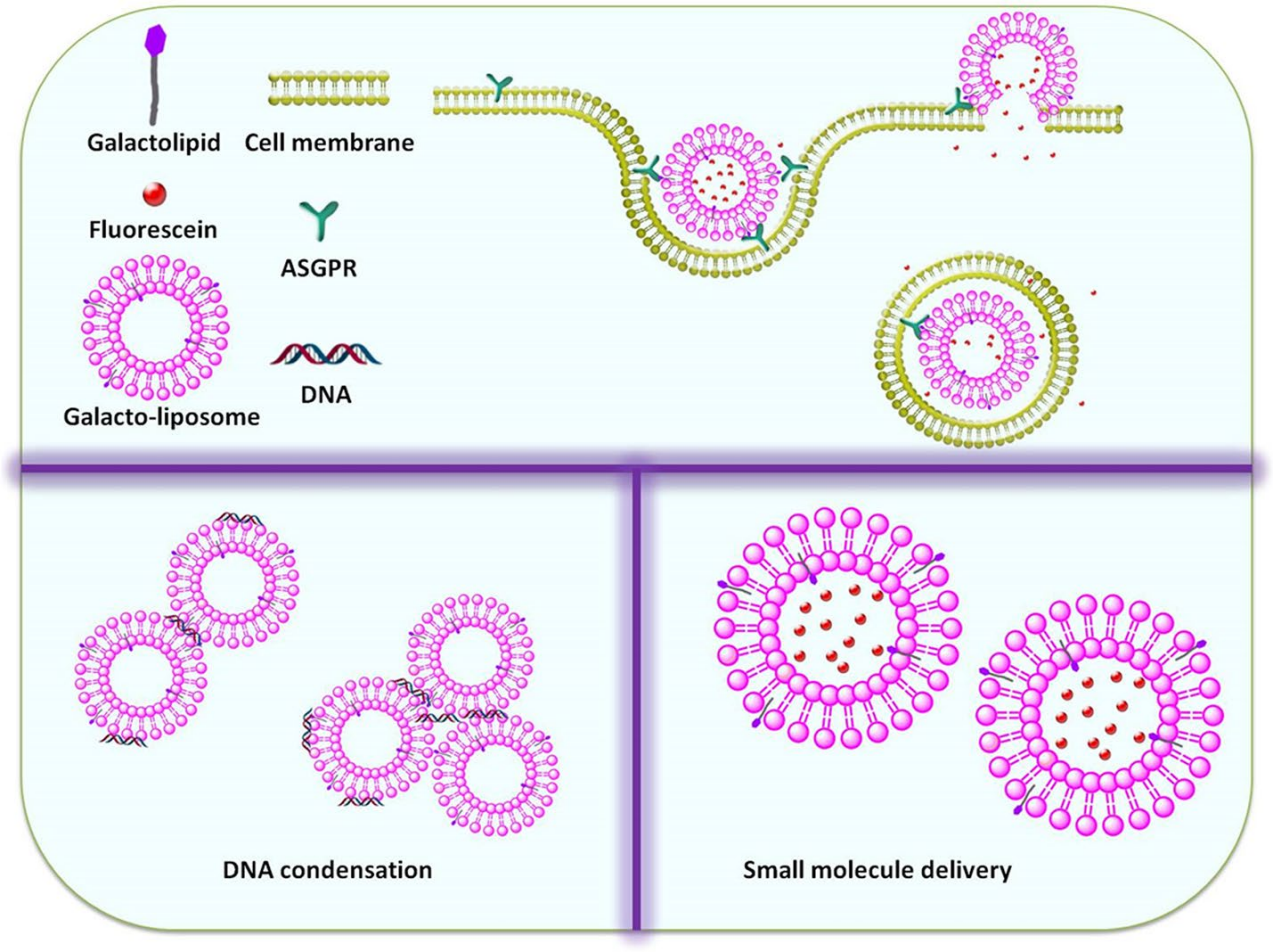
Structure of anomeric galactolipids incorporated liposomes with the ability to target huh 7

We had studied and reported on interfacial behaviors of anomeric galactolipid in DPPE monolayer prepared by LB technique. The interaction between molecules and molecular arranging manner were found to be depended on the ratios of galactolipid (Song et al. 2012). Here we use a pair of α-(**1**) and β-galacolipid(**2**) (Fig. 1) to investigate comparatively their liposomal properties of a DPPE-based liposome (Fig. 2). The dependencies and relationships between their thermo-stability/membrane fluidity/membrane density and the biofunctions, such as drug leakage efficiency and DNA condensation efficiency have been investigated. What kind of membrane might benefit the drug or DNA delivery has been discussed.

## Methods

### Preparation of liposome

5.0 mg of DPPE (99 %, Lipoid, Germany) and 1.0 mg of cationic cholesterol (Sigma, USA) (molar ratio ≈ 3/1) were dissolved in 5 mL of chloroform, and 2.5 mg of galactolipid was dissolved in 5 mL of dichloromethane containing minute quantity of methyl alcohol. Then the two solutions were mixed with different molar ratios. The organic solvents were removed by a rotary evaporator for 45 min at 37 °C and another 1 h under vacuum at room temperature. The lipid film was hydrated with ultrapure water (for characterization experiments) or Tris–HCl buffer solution (10 mM, pH 7.4, for biology experiments), and the suspension was sonicated in a bath sonicator under 65 °C for 30 min. The suspension was then extruded (11 times) through a polycarbonate membrane with a pore diameter of 200 nm (Whatman, UK) by using LiposoFast (LF-1, Avestin, Canada). The particle sizes were about 150 ± 10 nm measured by a dynamic light scattering (Zetasizer Nano S, Malvern, UK). Their phase transition temperatures were determined on a differential scanning calorimetry DSC (Setaram DSC III, USA) at a scan rate of 1 °C/min covering a temperature range of 25–70 °C. For TEM measurement, the samples were dropped onto a copper net and the morphologies of liposomes were recorded with JEOL JEM-1400.

### Membrane fluidity of liposome

The membrane fluidity of liposome was determined at 25 °C by a fluorophotometer (Perkin Elmer, LS55). 1.864 mL of 1.0 mg/mL liposome suspension was incubated with 2.5 mL of 2 μM DPH in water and then diluted to 5 mL at 25 °C for 60 min. Then the fluorescence (FL) intensity of DPH was measured at excitation and emission wavelengths of 360 and 425 nm, respectively. The values of FL polarization (*P*) were calculated using an analysis software (FL Winlab, Perkin Elmer, Co.) from the fluorescent intensity of 1,6-phenyl-1, 3, 5-hexatriene (DPH, Sigma) according to Eq. (1) (Kobayashi et al. 2007); the reciprocal value of polarization (1/*P*) was defined as the membrane fluidity:

1 $$ P = (I_{VV} - GI_{VH} )/(I_{VV} + GI_{VH} ) $$

where *I*_VV_ was the FL intensity measured with both excitation and emission polarized vertically, and *I*_VH_ with the vertically polarized excitation and horizontally polarized emission. The *G*-factor (*G*) was determined by the equipment as an impact factor, which is equal to *I*_HV_/*I*_HH_.

### Load and release of fluorescein

Liposomes were prepared as mentioned above except that the lipid membrane was hydrated with a fluorescein (400 μM) contained Tris–HCl buffer solution (10 mM, pH 7.4). The not-incorporated fluorescein molecules were removed by gel permeation chromatography on a Sephadex G50 resin column, which was preconditioned with PBS (pH 7.4). The fluorescein left in gel column was measured for calculating the loading amount in gel layer. The weight ratio of incorporated fluorescein to lipid was calculated to be 1/100 (g/g). 2 mL of fluorescein loaded liposome was sealed in a dialysis tube (MWCO 3500), which was placed in 30 mL of phosphate buffered saline (PBS, pH 7.4). The fluorescein leakages were investigated at 37 °C by using a spectrfluorometer (F-4500, Hitachi, Japan). The emission and excitation wavelengths were 517 nm and 495 nm. Complete release of fluorescein was obtained from controlling ethanol-treated liposome. The accumulated leakage percentage was then determined by the FL intensity relative to 100 % dequenching.

### Preparation of plasmid DNA

Plasmid DNA pIRES2-EGFP (pDNA) was purchased from BD Biosciences Clontech, USA. EndoFree Plasmid Maxi Kit (Qiagen, Germany) was used to prepare high-quality endotoxin free plasmid. Briefly, pDNA was transferred into competent cell *Escherichia coli* DH5α and streaked on kanamycin plate. A single colony was picked from the freshly selective plate and inoculated a starter culture of 3 mL Luria–Bertani medium containing 50 μg/mL kanamycin for 6 h at 37 °C with 300 rpm shaking. The starter culture was diluted at 1/500 into 200 mL LB medium containing 50 μg/mL kanamycin, and grew at 37 °C for 12 h with vigorous shaking (300 rpm). After inoculation, the bacterial cells were harvested by centrifugation at 4000×*g* for 15 min at 4 °C. After being re-suspended, lyses and neutralization, the pDNA was released from the lysate. Being recovered and purified with QIAGEN-tips, the pDNA was eventually resolved in 500 μL endotoxin-free Buffer TE at a final concentration of 1.2 μg/μL.

### Evaluation of pDNA condensation efficiency of liposome

pDNA was diluted with Tris–HCl buffer (pH 7.4) and mixed with liposome suspension at a terminal final concentration of 3.6 μg/mL. The mixture was kept for 30 min at room temperature before further experiment. The amount of pDNA protected from intercalation of EtBr by liposome was evaluated by ethidium intercalation assay (EtBr was excited at 520 nm to produce a FL emission at 595 nm). The pDNA composition efficiency was obtained through dividing the relative FL intensity of EtBr added to pDNA encapsulated in liposome by the maximum FL intensity of which added to free pDNA.

### In vitro cellular internalization effect of liposome to Huh7 cells

Rhodamine 6G loaded liposomes were prepared as mentioned above. Hepatocarcinoma cell line Huh7 was recovered from liquid nitrogen using DMEM (Gbico, USA) culture media with 10 % FBS and P-S antibiotics, and passed 2–3 generations before use. Cells were placed in a volume of 500 μL growth medium without antibiotics per well in a 24-well plate 1 day before transfection. The cells would be 90–95 % confluent at the time of transfection. The liposome suspension (30 μg/mL, 200 μL/per well) was transfected to 24-well plate containing 90–95 % confluent fresh cells and was rinsed by growth medium after 15 or 30 min incubation individually. Then the samples were imaged under a FL microscopy.

## Results

### Characterization of galacto-liposomes

Isomerism has been suggested to impact the LB monolayer property of glycolipids as well as the cell uptake of glycopolymer-containing nanoparticles (Liu et al. 2015). We have recently determined that anomerism, which is a common identity of nearly all glycoconjugates, could influence largely the bioactivity of glycolipids (He et al. 2011). Despite the extensive efforts in the construction of glyco-liposomes, investigations as regards the impact of anomerism on glyco-liposomes have been elusive.

Here we used a pair of anomeric galactolipids with a 16-carbon lipid chain, synthesized in a previous study (He et al. 2011), to test their liposomal properties while imbedded in a phospholipid-based liposome (Fig. 2). Liposomes that consist of pure DPPE (**LipoDPPE**), DPPE embedded with α-galactolipid **1** (**Lipo1**) or DPPE embedded with β-galactolipid **2** (**Lipo2**) of a similar particle size were fabricated. The mixing ratio was below 30 % as a previous study has suggested (Ueno et al. 2007) that further over-mixing would compromise the stability of glyco-liposomes.

**Fig. 3:**
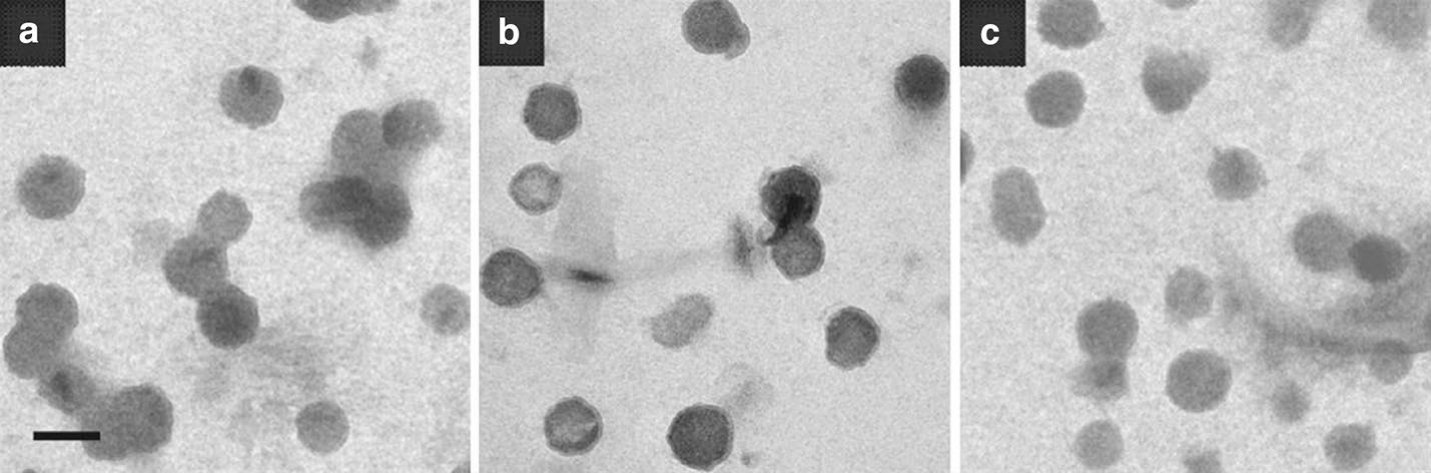
Typical scanning electron microscope image of **a** **LipoDPPE**, **b** **Lipo1** and **c** **Lipo2** (20 % galactolipid fraction). *Bar* 200 nm

The sizes and the *Zeta*-potentials of pure DPPE and galactolipid-incorporated liposomes were measured. The galacto-liposomes containing 10, 20 or 30 % of galactolipids were used for investigation. Their average sizes were determined to be 150 ± 10 nm (PDI: 0.15) after filtration by an extruder equipped with a polycarbonate membrane (pore diameter: 200 nm). Figure 3 shows the typical TEM images of the liposomes, indicating the doping of galactolipid did not give any influence on shape of liposomes. Their *Zeta*-potential was identically 45 ± 2 mV irrespective of the glycolipid doping ratio. The plenty of charge give the liposome suspension better stability to avoid the assembly.

**Fig. 4:**
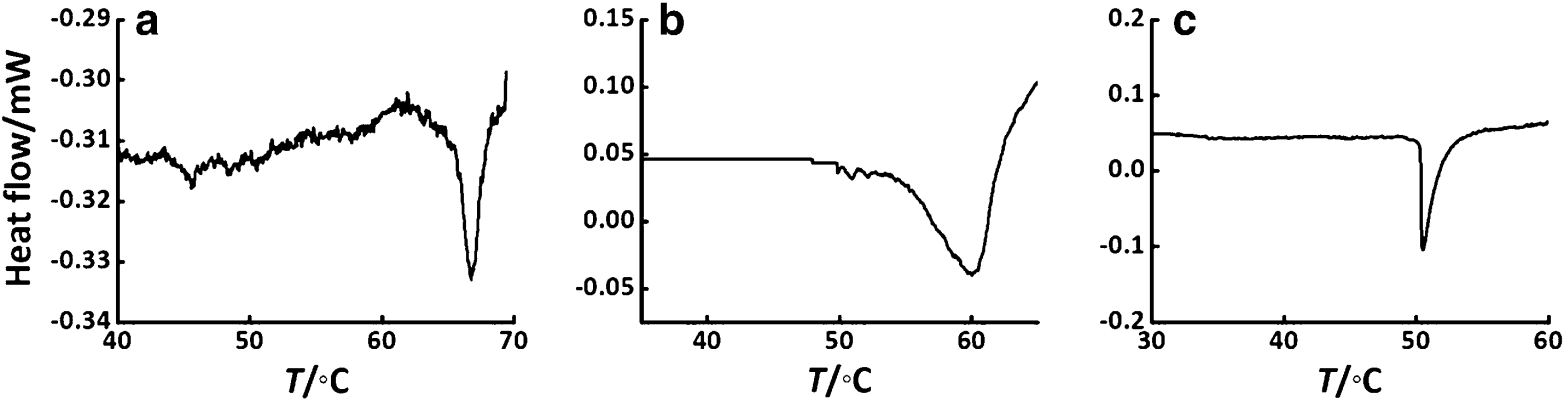
Typical DSC curve of **a** **LipoDPPE**, **b** **Lipo1** and **c** **Lipo2** (20 % galactolipid fraction)

**Fig. 5:**
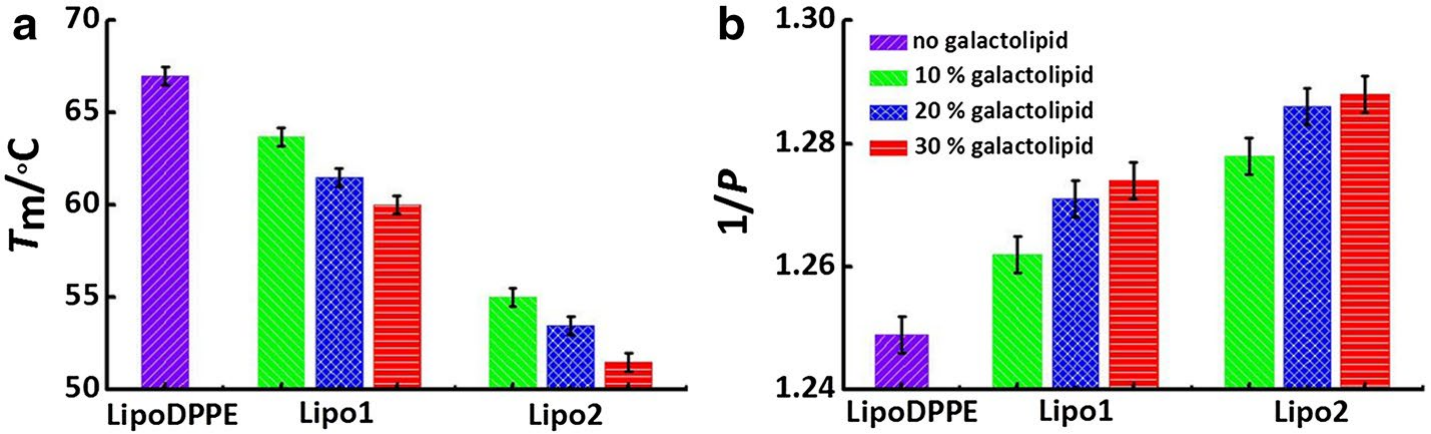
**a** *T*_m_ depicting the thermal stability (*T*_m_ of pure DPPE has been reported to be 64 °C) (Seddon et al. 1983), **b** 1/*P* values depicting the fluidity of **LipoDPPE**, **Lipo1** and **Lipo2** (10, 20 or 30 % galactolipid fraction)

The phase transition temperature (*T*_m_) of these liposomes were measured by using a DSC to test their thermo-stability. The typical DSC data shown in Fig. 4 suggests different decreases in *T*_m_ after imbedding the anomeric galactolipids into the DPPE liposome. Figure 5a shows a complete *T*_m_ comparison among the liposomes. The *T*_m_ value of **LipoDPPE** was measured to be 67 °C, which is similar to a literature report (64 °C) (Sun et al. 2014). In contrast, it decreased gradually with the increase of the doping ratio of galactolipids. The *T*_m_ values decreased to 60 and 52 °C for **Lipo1** and **Lipo2**, respectively, which suggests that the thermal stability decreases when the galactolipids are present.

The membrane fluidity of liposomes is an important parameter, which can influence liposomal biofunctions such as drug encapsulation and drug release. Indeed, higher membrane fluidity can result in a quicker drug leakage or releasing. The 1/*P* values, which are proportional to the membrane fluidity of the liposome, are calculated from the DPH FL polarizations (Fig. 5b) (Treichel et al. 1994). We observed that the 1/*P* values of **LipoDPPE** increased with increasing galactolipid, while the 1/*P* value of **Lipo1** was lower than that of **Lipo2**.

### The drug leakage of liposomes

**Fig. 6:**
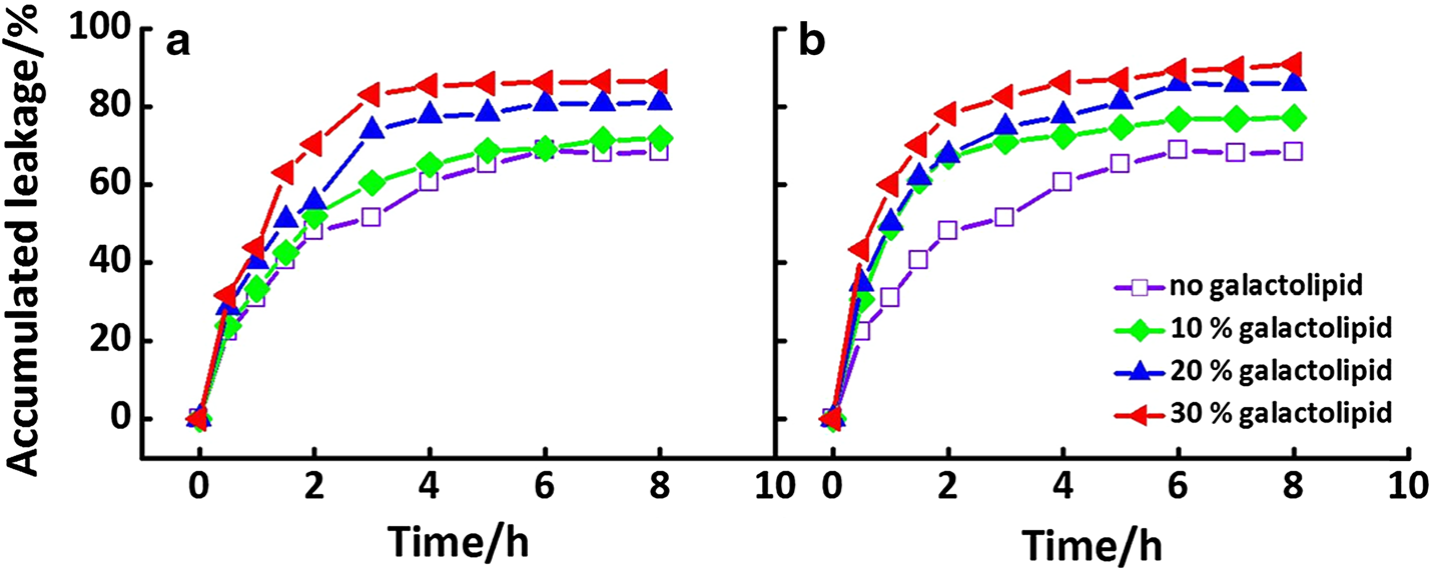
Dynamics curves of fluorescence leakage from galacto-liposomes, **Lipo1** (**a**) and **Lipo2** (**b**). The results of pure liposome were also shown in both

The stability of liposome could also be evaluated by the accumulated leakage of incorporated FL from various liposomes. The dynamics curves of FL release were obtained by measuring the FL intensity at regular intervals (Fig. 6). As expected, it showed an increase tendency with increasing molar ratio of galactolipid, agreeing with the results of 1/*P*, as shown in Fig. 5b. The accumulated leakages of **Lipo2** were slightly higher than those of **Lipo1**, indicating less stable membrane of the former.

### DNA condensation efficiency

The DNA condensation efficiencies of the pure liposome and galacto-liposome were judged through the FL intensity of EtBr. When DNA molecules were encapsulated or condensed, EtBr cannot insert into DNA base pairs and then fluoresce. A plasmid DNA that encodes the internal ribosome entry site and the enhanced green fluorescent protein (pIRES2-EGFP) was employed.

**Fig. 7:**
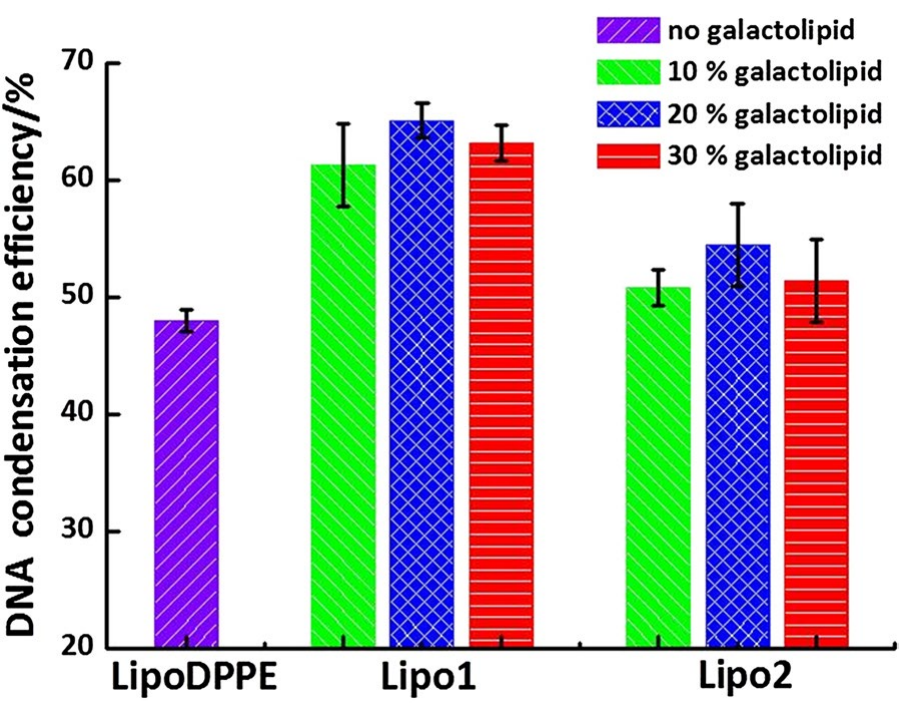
DNA condensation efficiency of **LipoDPPE**, **Lipo1** and **Lipo2**

Primarily, the size and *Zeta*-potential of the DNA-liposome complex were measured. Unlike the liposomes without DNA, the sizes of galacto-liposome increased a lot. The sizes of **Lipo1** (3000–3500 nm) increased more than those of **Lipo2** (1200–1500 nm), suggesting the attachment of DNA to liposome and, probably, formation of some aggregations. We observed that the efficiency of the anomeric galacto-liposomes was better than the pure DPPE liposome, whereas that of **Lipo1** superior to **Lipo2** (Fig. 7). And, the results also showed the DNA condensation changed a little with galactolipid ratios. Their variation trend was not like the results of *T*_m_ and 1/*P*, but a slightly maximum appeared at the doping ratio of 20 %.

### In vitro cellular internalization

**Fig. 8:**
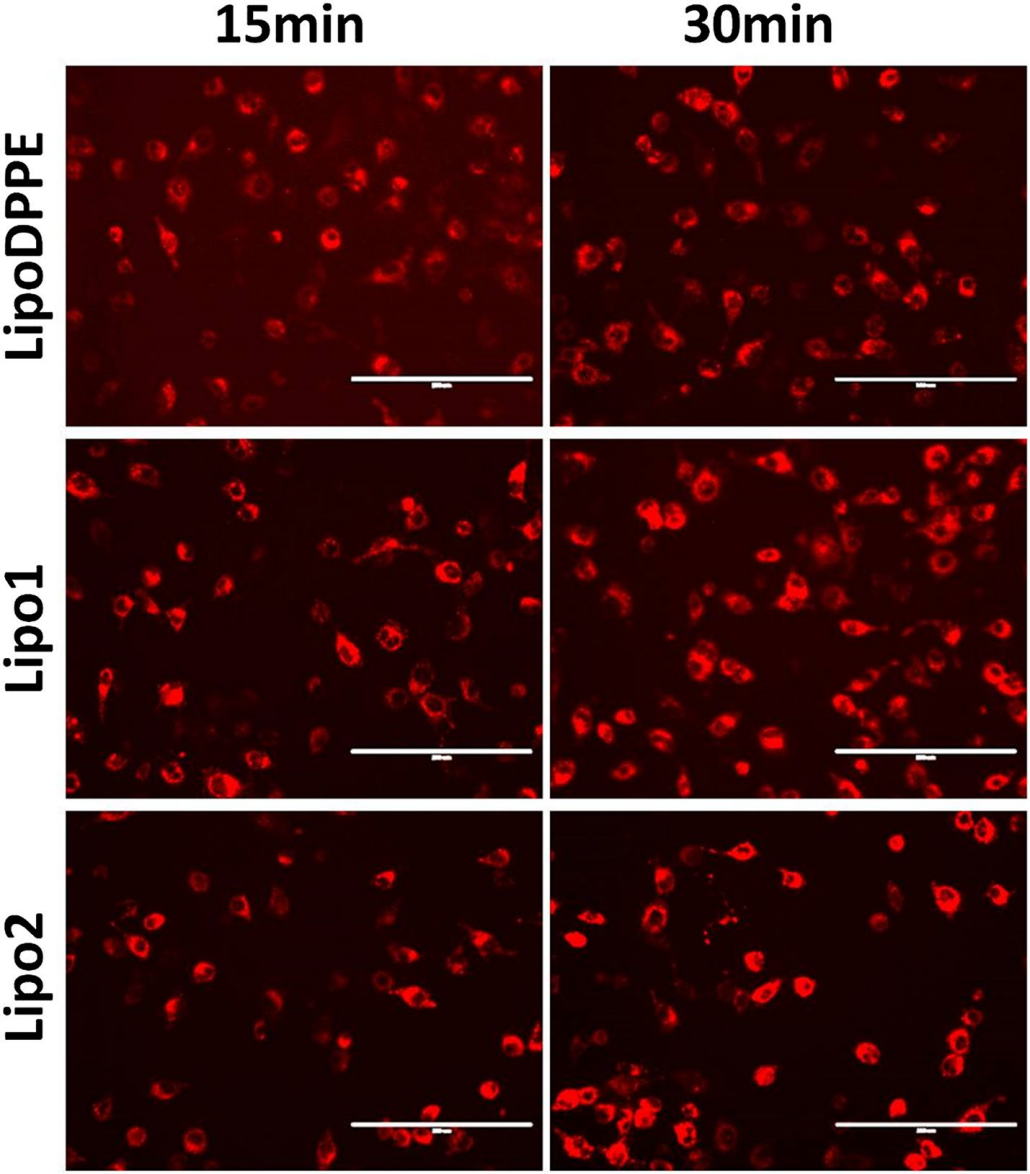
Fluorescence imaging of Huh7 cells (*scale bar* 200 μM) after transfection by **Lipo** **DPPE**, **Lipo1**, **Lipo2**. Culturing time: *left* 15 min, *right* 30 min (10 % galactolipid fraction)

**Fig. 9:**
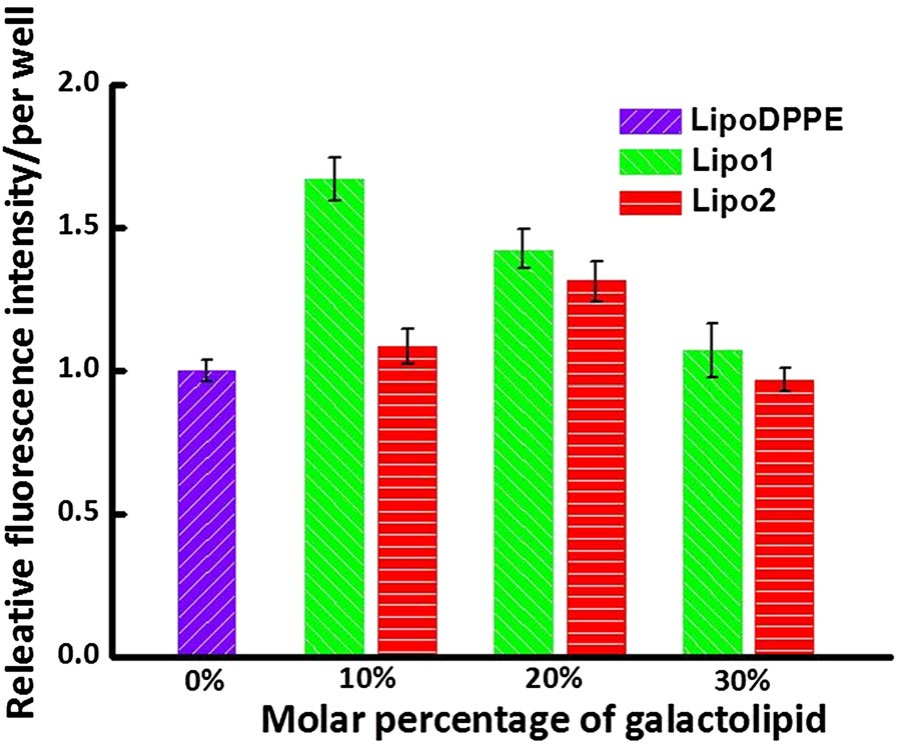
Releative fluorescence intensity of per well after 30 min incubation with **LipoDPPE**, **Lipo1** and **Lipo2**

The degree of cellular internalization of liposomes is a key parameter when used as drug/gene carrier. Here, the cellular internalization of these liposomes (with or without 10 % of galactolipid) towards a hepatoma cell line, Huh 7 that expresses ASGP-R, was evaluated. Cells were incubated with the liposomes for 15 or 30 min and then the FL was imaged (Fig. 8). Both anomeric galacto-liposomes (Fig. 8, **Lipo1** and **Lipo2**) were observed to show somewhat stronger FL than the pure DPPE liposome (Fig. 8**LipoDPPE**). Comparing the two galactolipid-liposomes, **Lipo1** containing α-galactolipid showed no obvious difference from **Lipo2** containing β-galactolipid judged from the FL images. Then a quantitative results of liposomes embedded with various ratios of galactolipid were shown in Fig. 9 with **LipoDPPE** used as control.

## Discussions

As galactolipid was incorporated into liposomes, the influence of the galactolipid on liposomes was characteries by DSC, FL polarization and drug leakage experiment. As shown in Fig. 5a, obviously, the imbedding of **2** has a larger impact on the stability of the liposomal property than **1**. It is known that imbedding lipids with unsaturated carbon bond or with shorter carbon chain should decrease *T*_m_ values and then thermo-stability. Here, being imbedded into the DPPE liposome, the anomeric galactolipids could probably have different interactions with DPPE which results from their small configurational difference as mentioned in a previous paper (Liu et al. 2015). The observation that the *T*_m_ of **Lipo1** is higher than **Lipo2** (Fig. 5a) might be caused by a stronger intermolecular interaction of the α-galacto-liposome than the β-counterpart, leading to the increased thermal stability.

From Fig. 5b, the 1/*P* values of **LipoDPPE** were found to increase with content of galactolipid, suggesting that the DPPE molecules are disturbed by addition of the galactolipid molecules. And **Lipo1** had lower 1/*P* value than **Lipo2**, which is also thought to be related to the interaction between DPPE and the anomeric galactolipids. A stronger interacton might hamper the motion of the moleclues in the bilayer.

For the fluorescein leakage experiment shown in Fig. 5b, their accumulated leakages increased with content of galactolipid and 1/*P* value. The accumulated leakages of **Lipo2** were slightly higher than those of **Lipo1**, indicating the less stable membrane of the former. As a drug carrier, high drug leakage is thought to increase side-effect of drug during cycling in body. However, too stable liposome might have another problem, that is, might tend to hardly release even if the carrier gets to the sick site. So it is necessary to study on drug release system which could release smartly and controllably.

From these results, we observed that, with the increase of the molar fraction of galactolipids, the thermal stability decreased and the fluidity of the liposome membrane increased. The higher membrane fluidity may account for its lower thermal stability. This is because the two properties might both relate directly to the motion of lipid carbon chain.

The ability of these liposomes to condense DNA was also investigated. Although it has been suggested that the charge amount, fluidity of membrane and alkyl chain length of lipsome had impact on the DNA condensation and transfection efficiency, the exact mechanisms are not known (Lentz 1993). It is easy to think that the DNA condensation efficiency should firstly depend on the *Zeta*-potential and size of the liposome, which have been unified in this experiment. And secondly, the micro-property of liposomal membrane, including membrane fluidity, intermolecular interaction, molecular density, etc., should be concerned. According to the discussion mentioned above, we focus on the first two. The difference in DNA condensation between **Lipo1** and **Lipo2** could probably be ascribed to the slightly higher fluidity and weaker molecular interaction of the β- than the α-galacto-liposome (Song et al. 2012), which is unfavourable to condense the DNA strand more efficiently. We deduced that the liposomal membrane with higher fluidity (**Lipo2**) might hamper the process of DNA adsorption and condensation, leading to decreased DNA condensation efficiency.

As for the cellular internalization ability of these liposomes, within the short incubation with cells, there was very little amount of rhodamine released from liposomes. So it could be thought that there was very little free rhodamine could enter into cells through permeabilization. The intensity of FL was used to judge the amount of internalized liposomes. It was found that the difference between the results of **Lipo1** and **Lipo2** was not significant from the FL images. However, both anomeric galacto-liposomes showed better internalization ability than the pure DPPE liposome, suggesting that the presence of the galactoside warhead might give an improvement in the function of cellular internalization.

From the quantitative results of internalization, it was found that increase the ratio of galactolipid did not improve the internalization linearly, suggesting this bioprocess of cellular internalization should be very complex. But we could confirm that it was impacted seriously by membrane properties. An appropriate fluidity of liposomal membrane should be necessary. It was indicated a lower containing ratio of galactolipid seemed to get a less drug leakage and a better internalization effect.

We note that obvious difference was observed between the anomeric counterparts (**Lipo1** and **Lipo2**), suggesting that further sophisticated experiments are needed to elaborate such difference. However, the above-shown cellular data clearly demonstrated the advantage of using **Lipo1** for preparing hepatoma cell targeted drug carriers.

## Conclusions

As what discussed above, using a pair of anomeric galactolipids we showed that their liposomal properties and biofunctions, while incorporated with a DPPE based liposome, were different as a result of their small configurational difference between α- and β-galacolipid. Galacto-liposomes (**Lipo1** and **Lipo2**) showed decreased thermal stability and increased membrnane fluidity, leading to the better DNA condensation efficiency probably due to stronger ability to bind and condense DNA chains. And also the all galacto-liposomes, especially **Lipo1**, showed better internalization ability than the pure DPPE liposome. We suggest that the elaboration of the structural and functional distinction between isomeric glyco-liposomes should be an important task to address in the field of targeted drug delivery, shedding light on the better understanding of the endocytosis and membrane fusion of these vesicles on the cellular level.
